# Dynamics of Thermolysis and Skin Microstructure in Water Buffaloes Reared in Humid Tropical Climate—A Microscopic and Thermographic Study

**DOI:** 10.3389/fvets.2022.871206

**Published:** 2022-05-25

**Authors:** Reíssa Alves Vilela, José de Brito Lourenço Junior, Manuel Antonio Chagas Jacintho, Antonio Vinícius Correa Barbosa, Messy Hannear de Andrade Pantoja, Carlos Magno Chaves Oliveira, Alexandre Rossetto Garcia

**Affiliations:** ^1^Institute of Veterinary Medicine, Federal University of Pará, Castanhal, Brazil; ^2^Laboratory of Skin and Leather Quality Assessment, Embrapa Southeast Livestock, Brazilian Agricultural Research Corporation, São Carlos, Brazil; ^3^Cyberspace Institute, Federal Rural University of the Amazon, Belém, Brazil; ^4^Laboratory of Biotechnology and Animal Reproduction, Embrapa Southeast Livestock, Brazilian Agricultural Research Corporation, São Carlos, Brazil

**Keywords:** acclimatization, *Bubalus bubalis*, heat stress, histology, infrared thermography, thermoregulation, precision livestock farming

## Abstract

The thermolytic capacity test is used to assess the adaptability of animals to existing environmental conditions. However, there is insufficient information on the relationship between histomorphometry and adaptability of buffaloes. Thus, this study aimed to assess the use of thermolysis pathways by buffaloes reared in a hot and humid environment so as to understand the relationships between environment, skin morphological characteristics, and heat storage, as well as the intensity and proportionality of use of its ways of dissipating heat to maintain homeothermy. The heat tolerance test, associated with the evaluations *via* infrared thermography, was applied to 10 female Murrah buffaloes and tegument histomorphometry was carried out. The animals exhibited very high heat tolerance with an average of 9.66 ± 0.21 and used thermal polypnea as the main heat dissipation pathway. Their mean skin thickness was 6.03 ± 1.16 mm and the active sweat and sebaceous gland tissue were 1.57 ± 0.38% and 1.08 ± 0.39%, respectively. The buffaloes exhibited a positive correlation between eyeball temperature and internal body temperature (*r* = 0.84523, *p* < 0.0001) and a negative correlation between respiratory rate and skin thickness (*r* = −0.73371, *p* = 0.0157). The high thermolytic capacity in shade conditions confirms the importance of access to shade in buffalo rearing systems in tropical regions.

## Introduction

Domestic buffaloes (*Bubalus bubalis*) are animals that inhabit different ecosystems (1). The worldwide buffalo population is estimated at approximately 208 million animals, 62% of which reared in the intertropical zone, distributed across 37 countries located in Asia (61.42%), Americas (1.16%), Africa (0.002%), and Oceania (0.002%) (2). There is also a considerable buffalo population that, despite being reared in subtropical or temperate climates, has been stricken with more intense and more frequent heat loads (3). In all these countries, one of the most important roles of buffaloes is, undoubtedly, milk production, significantly contributing to food safety, in addition to being efficient as draft power (2, 4). Thus, the buffalo is a model of multipurpose bovid which serves larger-scale productions as well as plays an essential role in the assets and economy of smallholders in several countries in Asia and Latin America, such as the Philippines, Thailand, Brazil, and Mexico (5–8). One of the most notable characteristics of buffaloes is their adaptative capacity (9). However, despite their adaptability to different environmental conditions, the water buffaloes have morphological peculiarities that impact the regulation of their body temperature (BT) (10, 11), making them more susceptible to heat stress, particularly when submitted to environmental conditions of high temperature and relative humidity and/or intense direct solar radiation (12).

The skin is the largest organ in the body and has a role of thermoregulation, defense, and protection of the organism (13). Buffaloes have thick skin, with a prominent stratum corneum, that can be twice as thick as the skin of bovines (11 μm vs. 5 μm) (14, 15). Besides very pigmented skin, the high melanin concentration provides them increased protection against ultraviolet radiation (10). Moreover, the epidermis of buffaloes extends through numerous papillae, which influences the distribution of blood vessels and favors the vasodilation process (16). However, the water buffaloes have a lower number of hair follicles (HF) on their body surfaces when compared with Zebu cattle (135–145 vs. 3.000 follicles.cm^−2^) (17) and this reduced the layer of reflecting fur on the epidermis makes them more susceptible to visible and infrared radiations, which are more absorbed and transmitted due to the black color of the epidermis (18, 19). Their hairs are relatively long and thick and connect to the skin associated with sweat and sebaceous glands (20). The density of sweat glands is low, and those glands are of the type apocrine and merocrine in a simple saccular and spiral tubular format, located deep into the reticular dermis and surrounded by blood vessels and nerve fibers (20). The sebaceous glands of buffaloes are simple or compound alveolar located in the reticular layer of the dermis and surround the entire HF, secreting sebum in a holocrine way (21).

Heat stress is antagonistic to animal welfare and leads to economic losses resulting from the reduction in productive and reproductive performance, besides increasing buffalo morbidity and mortality (22). The recent studies have shown the negative effects of heat stress on dairy buffaloes (23–25), with significant changes in milk composition and industrial yield such as reduced contents of fat, protein, lactose, and total solids (26–29). In addition, the negative effects of heat stress have been reported on conception rates, which decrease when temperature-humidity index values are above 80 (30–32), and on semen quality, with decreased percentage of live spermatozoa and increased abnormal sperm population (33–36). Heat stress also impairs the immune response, affecting the gene expression of cytokines and their receptors (37, 38). To mitigate the deleterious effects of heat stress in water buffaloes and provide them greater thermal comfort, establishing environmental management strategies (39) as well as the identification and selection of more heat-tolerant animals are equally important (40, 41).

A thermotolerant animal is able to maintain its homeothermy even under high environmental heat loads (29). Several measures have been proposed for phenotyping thermotolerant animals, which, in general, include functional evaluations based on the monitoring of physiological variables related to body thermoregulation, including internal temperature, respiratory and heart rates, and blood parameters. Among the blood or serum parameters commonly assessed are hematocrit, hemogram, hemoglobin concentration, oxidative stress markers, and the concentration of glucocorticoid and thyroid hormones and of heat shock proteins (42, 43). In turn, body surface temperature (ST) monitored by infrared thermography has been used as an indicator of heat production since this technique allows assessing the amount of thermal energy emitted by longwave radiation on a surface (19). Implementing a program for the identification and selection of thermotolerant animals requires evaluation strategies that can be adopted easily and with low cost. In this sense, the heat tolerance test (HTT) has been used to assess the adaptability of water buffaloes to hot environments (44), a test that requires relatively little time and can be performed on-farm (45). However, there are no studies associating buffalo thermolytic response (capacity of dissipating heat through peripheral vasodilation, sweating, and thermal polypnea) in this test with microscopic anatomic tegument characteristics, which is a gap in knowledge seen as the buffalo tegument system has features that completely distinguish it from other domestic animal species. Although some morphological particularities of buffaloes are known, there is a little fundamental knowledge on the histomorphometric characteristics of the skin of those animals and their relationship with thermolytic adaptive capacity. Therefore, this study is proposed aiming at expanding the knowledge and understanding of buffalo thermoregulating mechanisms and thermal transfer mechanisms between the animal and the environment. The aims of the study are as follows: To (i) assess the thermolytic responses of buffaloes submitted to thermal challenge in a humid tropical environment and (ii) determine the relationships between the thermal environment, cutaneous morphological characteristics, and heat storage (HS), as well as the intensity and proportionality of the use of heat dissipations pathways to maintain homeothermy.

## Materials and Methods

### Site, Climate, and Period

The experiment was conducted at the Biotechnology Center of Animal Reproduction—CEBRAN of the Federal University of Pará, in Castanhal, PA, Brazil (01°30'48”S and 47°94'23”W, 41-m altitude). The local climate subtype was humid tropical, Afi (Köppen), characterized by well-distributed annual rainfall, with a rainier period from January to June and a less rainy period from July to December. The mean annual air temperature was 26.6°C (min. 23.0°C; max. 32.0°C). The mean annual relative humidity was 83% and the cumulative annual rainfall was 2,900 mm (46). The study was conducted between November and December.

### Animals and Handling

Ten female Murrah buffaloes previously reared under the same environmental and handling conditions were used. Prior to the beginning of the study, the animals underwent clinical evaluation, diagnostic tests, and a quarantine period to ensure proper health condition. At the beginning of the experiment, the animals were 18 ± 0.7 months of age (18–20 months), with mean weight of 336.3 ± 38.2 kg (264–398 kg) and body condition score of 3.0 ± 0.4 (2.5–4.0) (scale from 1–5) (47). The buffaloes were kept in a 0.2-ha pen with *Brachiaria decumbens* pasture and natural shade (90 m^2^ per animal). The animals were fed daily (between 8 a.m. and 4 p.m.) supplementary feed made up of elephant grass (*Pennisetum purpureum*) silage and concentrate based on wheat bran, in addition to mineral mixtures. Access to the automated drinking trough was *ad libitum*.

### Heat Storage and Cumulative Heat Storage

The buffaloes were individually weighed on an electronic scale and the body weight was used to calculate HS (in W·m^−2^) and cumulative heat storage (CHS) (W·m^−2^ h^−1^) on the days of data collection, according to methodology described by McGovern and Bruce (48) [Eqs. (1) and (2), respectively].


(1) $$\begin{array}{r} \Delta RT=\left(\left(3600\times HS\times A\right)\right)/\left(\left(Bw\times cb\right)\right) \end{array}$$

where ΔRT is the differences between rectal temperatures at different hours; HS is the HT (W·m^−2^); A is the animal surface (m^2^) calculated by the equation: *A* = 0.13 × *Bw*^0.556^ (where Bw is the body weight in kg) and cb is the specific heat of the animal (3.4 KJ·kg^−1^ K^−1^).


(2) $$\begin{array}{r} CHS=\;\overset{3}{\underset{12}{\sum }}HS \end{array}$$

where CHS is the cumulative heat storage (W·m^−2^.h^−1^) calculated the by sum of the storage heat in different time intervals (from 12 p.m. to 3 p.m.), with the zero value being considered at 12 p.m.

### Heat Tolerance Test

To determine the individual thermolysis capacity, the heat tolerance test (HTT) described by Baccari Junior et al. (44) and modified by Titto et al. (45, 49) was applied. Over a 15-days period, the test was applied on 4 non-consecutive days, which exhibited specific and predetermined meteorological characteristics of cloudless sky, no rainfall or wind gusts, and minimum black globe temperature of 45°C in full sunlight. During the application of the test, the animals were kept standing to avoid heat exchange with the soil *via* conduction, and remained in food and water fast so as to not change HS (45).

For the test, the buffaloes were conducted to a corral with holding pens and rough concrete floor, 4-m ceiling, and roofing with ceramic tiles, which provided 162 m^2^ of shade, with access to an adjacent open area of 120 m^2^ with dirt floor, with no drinking or feeding troughs, and delimited by a fence with six smooth wires, where the animals were exposed to direct solar radiation.

According to the protocol of the HTT by Baccari Junior et al. (44), modified by Titto et al. (45, 49) to compare the rates of heat acquisition and dissipation, the buffaloes were kept under shade condition for 2 h, from 10 a.m. to 12 p.m. (Period 0). The physiological variables recorded in this period represented the baseline individual reference values. Next, the animals were exposed to solar radiation for 1 h, from 12 p.m. to 1 p.m. (Period 1), the period of heat challenge. After that, the animals were returned to the shade, where they were kept for 2 h, from 1 p.m. to 2 p.m. (Period 2) and from 2 p.m. to 3 p.m. (Period 3), representing the period for tolerance recovery of the animals after the heat challenge. By the end of Periods 1, 2, and 3, the physiological variables of internal body temperature, respiratory rate, and body ST were recorded (see description in the Section, Physiological variables). All buffaloes were assessed on the same days as the test.

Next, the heat tolerance index (HTI) was calculated based on the equation that takes into account the mean internal body temperatures in Periods 0 and 3 [Eq.(3)].


(3) $$\begin{array}{r} HTI=10-\left(BT3-BT0\right) \end{array}$$

where HTI is the heat tolerance index, which is calculated by the difference between the internal BT obtained at the end of Period 3 (BT3) and the internal BT obtained at the end of Period 0 (BT0).

The result of the HTI varies on a scale from 0 to 10, which represents the capacity of the animals to dissipate the heat absorbed during exposure to sunlight. The closer the result is to 10, the more tolerant the animal (44). According to Titto et al. (45), an animal is classified as very sensitive to heat stress when its HTI is below 7.5; low heat tolerance when its HTI is between 7.51 and 8.2; medium heat tolerance with HTI between 8.21 and 8.9; high heat tolerance with HTI between 8.91 and 9.5; and very high heat tolerance when its HTI is equal to or higher than 9.51.

### Physiological Variables

The comparison of the rates of heat acquisition and dissipation was based on alterations of internal BT (°C), respiratory rate (RR) (mov·min^−1^), surface temperature (ST) (°C), and on the calculation of the respective HS in each experimental period. RR was recorded *via* inspection and counting of thoracic movements for 1 min with the aid of a stopwatch and was recorded before the animals entered the holding pen for verification of the other physiological variables. The BT was measured by clinical transrectal thermometry using a digital thermometer (Termomed, Incoterm, Brazil) with the measuring range, 32.0–42.0°C, resolution of 0.1°C, maximum error of ± 0.2°C, and a self-checking system.

### The ST by Infrared Thermography

The ST was assessed by infrared thermography using a portable thermographic camera (FLIR E-5, Oregon, USA) operating in the spectral range of 7.5–13 μm, with detector of 160 × 120 pixels, thermal sensitivity of <100 mK, field of view of 45° × 34°, and an automatic calibration. The thermographic images were always recorded on the right-side antimere of the animals, with 0.98 emissivity adopted according to indications by Brcko et al. (50). All thermograms were analyzed in the software FLIR Tools (FLIR tools, RRID:SCR_016330).

**Figure 1:**
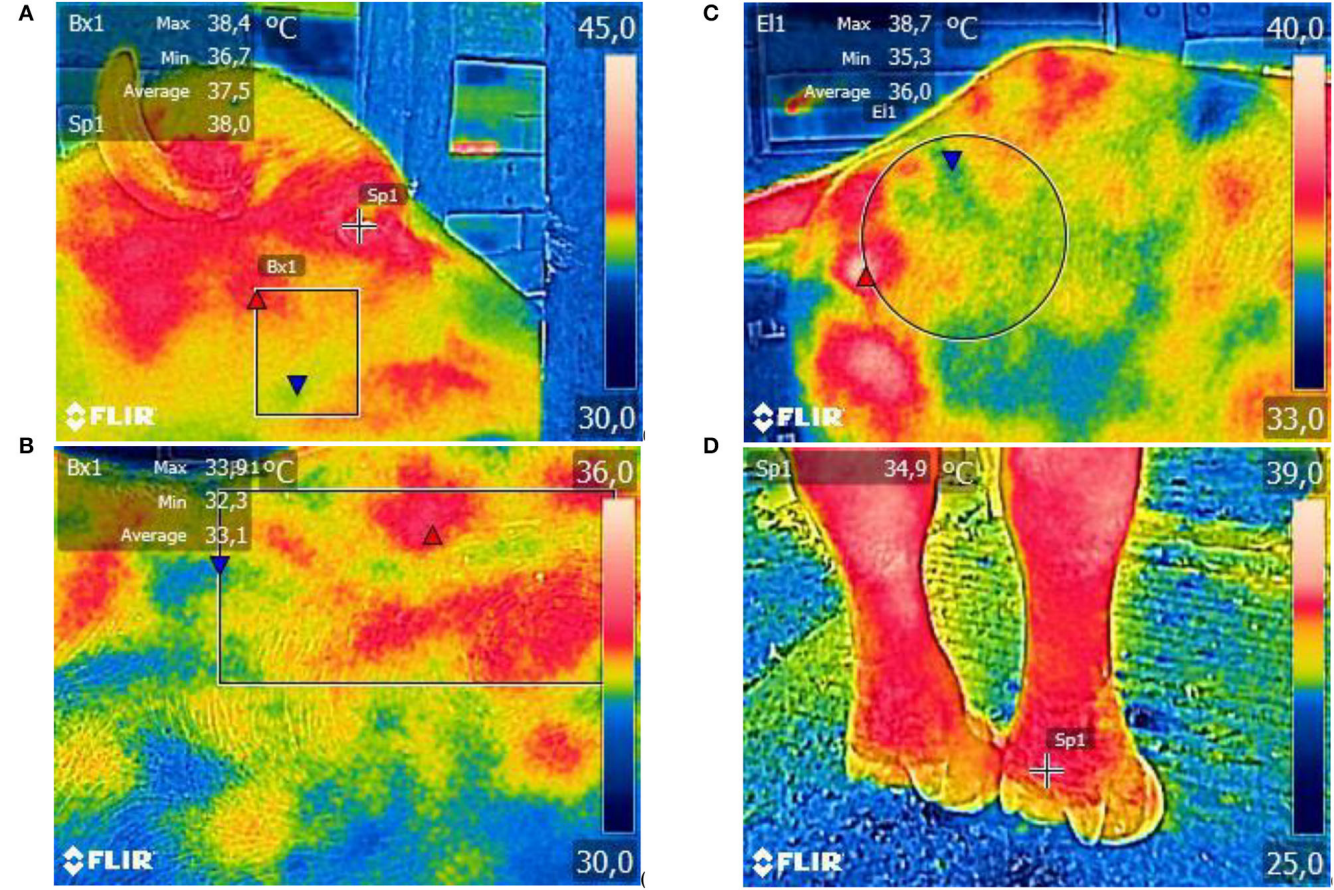
Illustrative images of thermographic images taken to assess STs of the eyeball and cheek **(A)**, trunk **(B)**, rump **(C)**, and forelimb **(D)** of adult female buffaloes. Images parametrized for the rainbow color palette and automatic thermal scale.

The STs of interest (Figure 1) were eyeball surface temperature (EST) (°C), resulting from the hotspot recorded in the eyeball region (51); cheek surface temperature (CST) (°C), determined by the mean temperature of the rectangular area between the region of the zygomatic and jaw bones (50); trunk surface temperature (TST) (°C), determined by the mean temperatures of the rectangular area in the dorsal region between the sternal ribs (50); rump surface temperature (RST) (°C), determined by the mean temperature of the circular area between the ileum and ischium (50); and forelimb surface temperature (FST) (°C), measured in the region of the ergot, above the phalanx (50). In addition, the compound surface temperature (CompST) (°C) was calculated, a derivative variable expressed by the arithmetic mean of the STs in the other body regions assessed, as proposed by Sevegnani et al. (52).

### Biometeorological Monitoring and Black Globe Temperature and Humidity Index

During the experiment, the biometeorological variables of dry-bulb temperature (DBT; °C), relative humidity (RH) (%), and black globe temperature (°C) were permanently monitored and recorded in 10-min intervals using electronic devices placed in the handling corral, in the environments where the animals underwent the HTT, in full sunlight, and in the shade. The variables were monitored using a HOBO U12-012 datalogger (Onset, Brazil) (HOBOware Pro, RRID:SCR_021915) installed at a height of 1.60 m from the ground. The BGHI was later calculated as described by Buffington et al. (53), determined by Eq. (4).


(4) $$\begin{array}{r} BGHI\;=T_{bg}+\left(0.36\times T_{dp}\right)+41.5 \end{array}$$

where T_bg_ is the black globe temperature (°C) and T_dp_ is the dew point temperature (°C).

According to Baêta and Souza (54), BGHI values up to 74 indicate a comfort condition for the animals; from 74 to 78, an alert situation; from 79 to 84, a danger situation; and above 84, an emergency situation.

**Figure 2:**
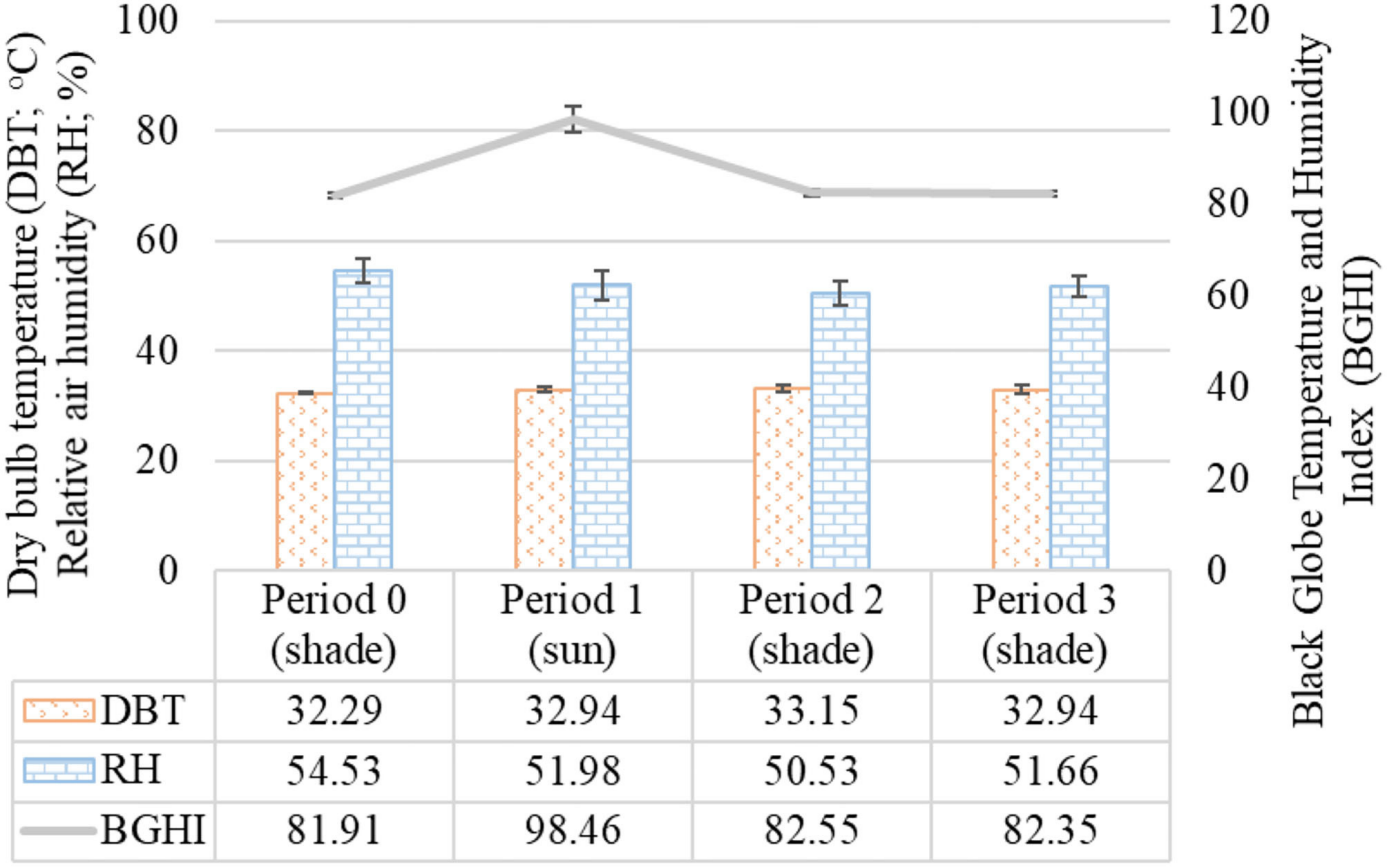
Mean and standard deviation of the biometeorological variables recorded in different observation periods during the HTT carried out in adult female buffaloes in humid tropical climate. Period 0 (shade): From 10 a.m. to 12 p.m.; Period 1 (sun): From 12 p.m. to 1 p.m.; Period 2 (shade): From 1 p.m. to 2 p.m.; Period 3 (shade): From 2 p.m. to 3 p.m.

The biometeorological data recorded during the HTT are presented in Figure 2.

### Skin Histomorphometry

Thirty days after the end of the HTT, the skin microbiopsy was performed in all animals for histomorphometric analyses. The microbiopsy was performed on the right-side antimere at the mean height of the back, in the region of the 12th intercostal space. The buffaloes were in water and food fast for 12 h and the microsurgical procedures were performed after physical restraining the animal and local skin anesthesia as described by Kahwage (55).

The samples were fixed in 10% formalin solution for 48 h and then underwent an 8 h cycle in an automated tissue processor (OMA DM-40, OMA Metalúrgica, Brazil) for dehydration and clarification. The samples were then included in paraffin blocks and processed histologically, with 4-μm thick cuts parallel and perpendicular to the skin surface made. Next, the samples were mounted on glass slides, stained using Masson's trichrome method (56), and assessed in light-field optical microscopy (Leica DME, Leica Microsystems, Germany) (Leica Microsystems, RRID:SCR_008960). The assessment was carried out with total magnification of 40× for the parallel cuts and 100× for the perpendicular ones. The images were digitized and stored in a database for later analysis using the software Image J (ImageJ, RRID:SCR_003070) (57).

**Figure 3:**
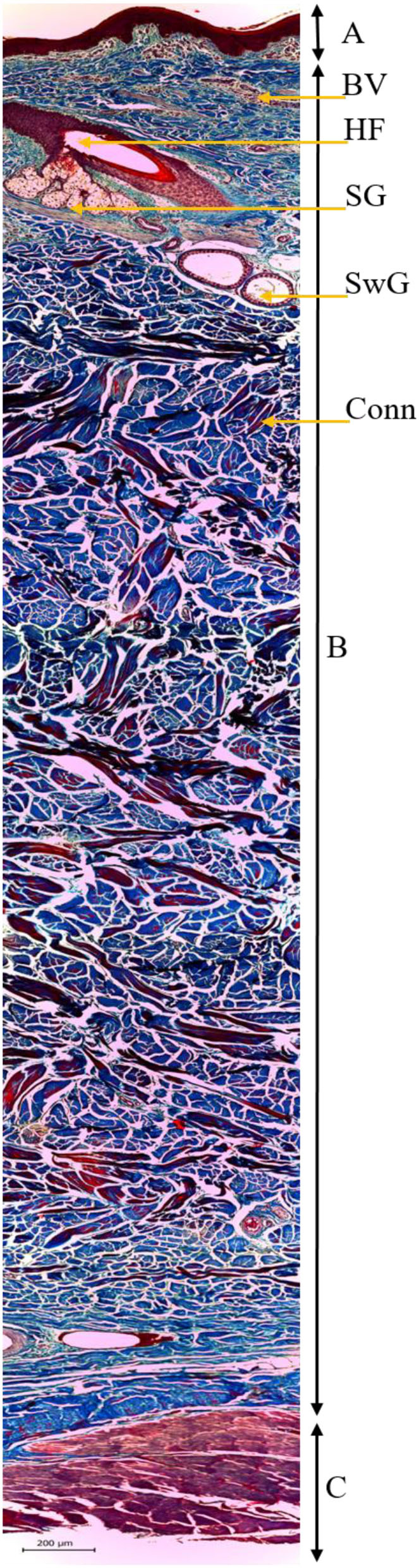
Histological image of the skin of buffaloes (*B. bubalis*) stained by Mason's trichrome, assessed in light-field optical microscopy (100×): epidermis **(A)**, dermis and hypodermis **(B)**, and smooth musculature **(C)**, with emphasis on the identification of the hair follicle (HF), sebaceous glands (SG), sweat glands (SwG), blood vessels (BV), and conjunctive tissue (Conn).

In the digitized images of the parallel cuts, the following parameters were assessed: (i) The number of HF; and (ii) hair density (HD) (follicles·mm^−2^). In the digitized images of the perpendicular cuts, the following parameters were assessed: (i) Thickness of skin (TS) (mm); (ii) sweat gland area (ASwG) (μm^2^), which corresponds to the mean value of the area of each sweat gland; (iii) sebaceous gland area (ASG) (μm^2^), which corresponds to the mean value of the area of each sebaceous gland; (iv) sweat gland area by surface (ASwG_surface) (%), which corresponds to the percentage of active sweat gland tissue in the sample; (v) sebaceous gland area by surface (ASG_surface) (%), which corresponds to the percentage of active sebaceous gland tissue in the sample; and (vi) height of sweat gland (HSwG) (μm) at four spots of the gland epithelium (58). The TS was determined after the mounting of composite images (Figure 3) using the software Adobe Photoshop CS5, with the standard of 30% overlap adopted between images to create homogeneous mosaics. Five composite images were analyzed per animal with three skin thickness measurements per image at three distinct anatomic spots. Skin thickness was given by the linear distance measured on the surface of the epidermis until the smooth musculature, with the mean value of each sample being adopted.

### Statistical Analysis

The response variables were approached using different statistical models according to the characteristic of each variable. Initially, a descriptive analysis of the data was made for the biometeorological and physiological variables. Data normality was evaluated by Shapiro–Wilk and/or Kolmogorov–Smirnov tests and the equality of variances was evaluated by Levene's test. For the biometeorological variables, one-way ANOVA (DBT and RH) and Kruskal–Wallis test (BGHI) were applied. For the parametric analyses of the physiological variables, fixed-effects (treatment-periods) and random-effects (animals and date) ANOVA were applied. The means between periods were compared by Tukey's test and, if needed, the data were transformed by BoxCox to meet the assumptions of the parametric analyses. All those analyses considered significance at 5%.

In addition, the factorial analysis by principal components (PCs) was performed to cluster and identify the behaviors of the correlations between the variables TS, ASG_surface, RR, CHS, and HTI. Bartlett's test of sphericity and Kaiser–Meyer–Olkin test were performed to assess whether the factorial analysis fitted the data. Finally, bivariate Pearson linear correlation analysis was carried out for the variables RR, BT, EST, CST, TST, RST, FST, CHS, BGHI, HF, HD, TS, HSwG, ASwG, ASwG_surface, ASG, ASG_surface, and HTI. The data were analyzed in the softwares SAS version 9.1.3 (Statistical Analysis System, RRID:SCR_008567) (59) and Rstudio version 1.4.1717 (R Project for Statistical Computing, RRID:SCR_001905) (60).

## Results

Based on the averages of the biometeorological variables, it was found that DBT (*p* = 0.13251) and RH (*p* = 0.12783) did not change during the observation times over the HTT. The mean maximum values of DBT were 33.15 ± 0.57°C with coefficient of variation of 1.72% recorded in Period 2. The mean maximum values of RH were 54.53 ± 2.16% with coefficient of variation of 3.96% recorded in Period 0. However, the non-parametric analysis by Kruskal–Wallis test indicated a significant difference for BGHI between the experimental periods (*p* = 0.02731), with the highest BGHI value of 98.46 ± 2.88 with coefficient of variation of 2.93% recorded in Period 1.

The ANOVA for the physiological and biophysical variance exhibited a significant effect (*p* < 0.0001) for all sources of variation and the means adjusted by least squares are presented in Table 1.

**Table 1:** Mean and standard deviation of the physiological and biophysical variables assessed in adult female buffaloes in the Eastern Amazon during the HTT.

**Effects**	**Period 0**	**Period 1**	**Period 2**	**Period 3**
**Variable**	**Mean ±SD**	**Mean ±SD**	**Mean ±SD**	**Mean ±SD**
BT	38.05 ± 0.11^c^	38.96 ± 0.16^a^	38.38 ± 0.12^b^	38.36 ± 0.12^b^
RR	22 ± 2.18^b^	81 ± 11.52^a^	22 ± 0.83^b^	20 ± 1.05^b^
EST	36.17 ± 0.68^c^	39.99 ± 1.07^a^	37.68 ± 0.61^b^	37.53 ± 0.45^b^
CST	35.37 ± 0.88^c^	39.89 ± 1.13^a^	36.62 ± 0.73^b^	36.33 ± 0.65^bc^
TST	34.68 ± 0.74^c^	40.59 ± 1.58^a^	36.04 ± 0.74^b^	35.92 ± 0.74^b^
RST	34.18 ± 0.71^c^	40.66 ± 0.89^a^	35.61 ± 0.98^b^	35.44 ± 1.38^b^
FST	33.66 ± 1.03^c^	39.97 ± 1.45^a^	35.66 ± 1.07^b^	35.08 ± 1.25^c^
CompST	34.82 ± 0.76^c^	40.23 ± 0.55^a^	36.33 ± 0.68^b^	36.06 ± 0.79^b^
HS	0^b^	0.0216 ± 0.0030^a^	−0.0138 ± 0.0032^c^	−0.0003 ± 0.0021^b^
CHS	0^c^	0.0649 ± 0.0091^a^	0.0233 ± 0.0123^b^	0.0222 ± 0.0117^b^

*Period 0 (shade): From 10 a.m. to 12 p.m.; Period 1 (sun): From 12 p.m. to 1 p.m.; Period 2 (shade): From 1 p.m. to 2 p.m.; Period 3 (shade): From 2 p.m. to 3 p.m*.

*BT, Internal body temperature (°C); RR, Respiratory rate (mov·min^−1^); EST, Eyeball surface temperature (°C); CST, Cheek surface temperature (°C); TST, Trunk surface temperature (°C); RST, Rump surface temperature (°C), FST, Forelimb surface temperature (°C); CompST, Compound surface temperature (°C); HS, Heat storage (W·m^−2^); CHS, Cumulative heat storage (W·m^−2^ h^−1^)*.

*^a−c^Means followed by equal lowercase letters within the row do not differ statistically by Tukey's test (p < 0.05)*.

The animals staying for 1 h under full sunlight, from 12 p.m. to 1 p.m., resulted in an increase in physiological variables related to thermolysis, with an increase by 0.91°C in BT, 59 mov·min^−1^ in RR, and 5.41°C in CompST, with the highest increase in ST recorded in the rump region at 6.48°C.

In period 2, a decrease at the same proportion was found, by 59 mov·min^−1^ in RR, which returned to baseline values, with no difference among Periods 0, 2, and 3. The same behavior of reduction in body STs was observed regardless of the anatomic region assessed. Two hours after the animals returned to the shade (Period 3), CST and FST returned to baseline values, reflecting in the reduction in internal body temperature, demonstrated by HS and CHS, with a decrease by up to 0.6°C in BT, with no difference between Periods 2 and 3.

The descriptive analysis of the histomorphometry variables and the HTI of the animals is presented in Table 2.

**Table 2:** Number of observations (*n*), averages ($\bar{X}$), standard deviation (SD) coefficient of variation (CV), minimum (Min.) and maximum (Max.) values for the heat tolerance index, and histomorphometric characteristics of the skin of adult female buffaloes reared in the Eastern Amazon.

**Variable**	**Unit**	***n***	** $\bar{\text{X}}$ **	**SD**	**CV**	**Min**.	**Max**.
HTI	-	40	9.66	0.21	2.15	9.20	10.00
HF	-	10	12.40	2.12	17.09	10.00	16.00
HD	hair·mm^−2^	10	2.00	0.26	13.11	1.59	2.41
TS	mm	10	6.03	1.16	19.21	4.77	8.28
HSwG	μm	10	16.20	1.99	12.28	12.00	19.00
AswG	μm^2^	10	17,283.9	4,449.8	25.75	13,102.1	26,450.9
ASwG_surface	%	10	1.57	0.38	24.16	1.14	2.30
ASG	μm^2^	10	11,821.9	4,301.9	36.39	4,834.0	19,175.8
ASG_surface	%	10	1.08	0.39	35.98	0.44	1.73

*HTI, Heat tolerance index; HF, Number of hair follicles; HD, Hair density; TS, Thickness of skin; HSwG, Height of sweat gland; ASwG, Mean value of the area of each sweat gland; ASwG_surface, Percentage of active sweat gland tissue in the sample; ASG, Mean value of the area of each sebaceous gland, ASG_surface, Percentage of active sebaceous gland tissue in the sample*.

The mean HTI recorded during the HTT was 9.66 ± 0.21, ranging from 9.20 to 10.00. For the histomorphometric variables, the averages of HD of 2.0 ± 0.26 mm^2^, TS of 6.03 ± 1.16 mm, HSwG of 16.2 ± 1.99 μm, ASwG of 17,283.92 ± 4,449.85, and ASG of 11,821.95 ± 4,301.90 μm^2^ were observed per animal. The percentage of active sweat gland tissue was 1.57 ± 0.38% and the percentage of active sebaceous gland tissue was 1.08 ± 0.39%.

The results obtained by the PCs technique, with the respective eigenvalues and percentages of variance explained by each one, show that the first two PCs accounted for 86.2% of the total variation on the thermolytic capacity of buffaloes, with principal component 1 (PC1) accounting for 45.2% and principal component 2 (PC2), for 41.0% of the data variance.

**Figure 4:**
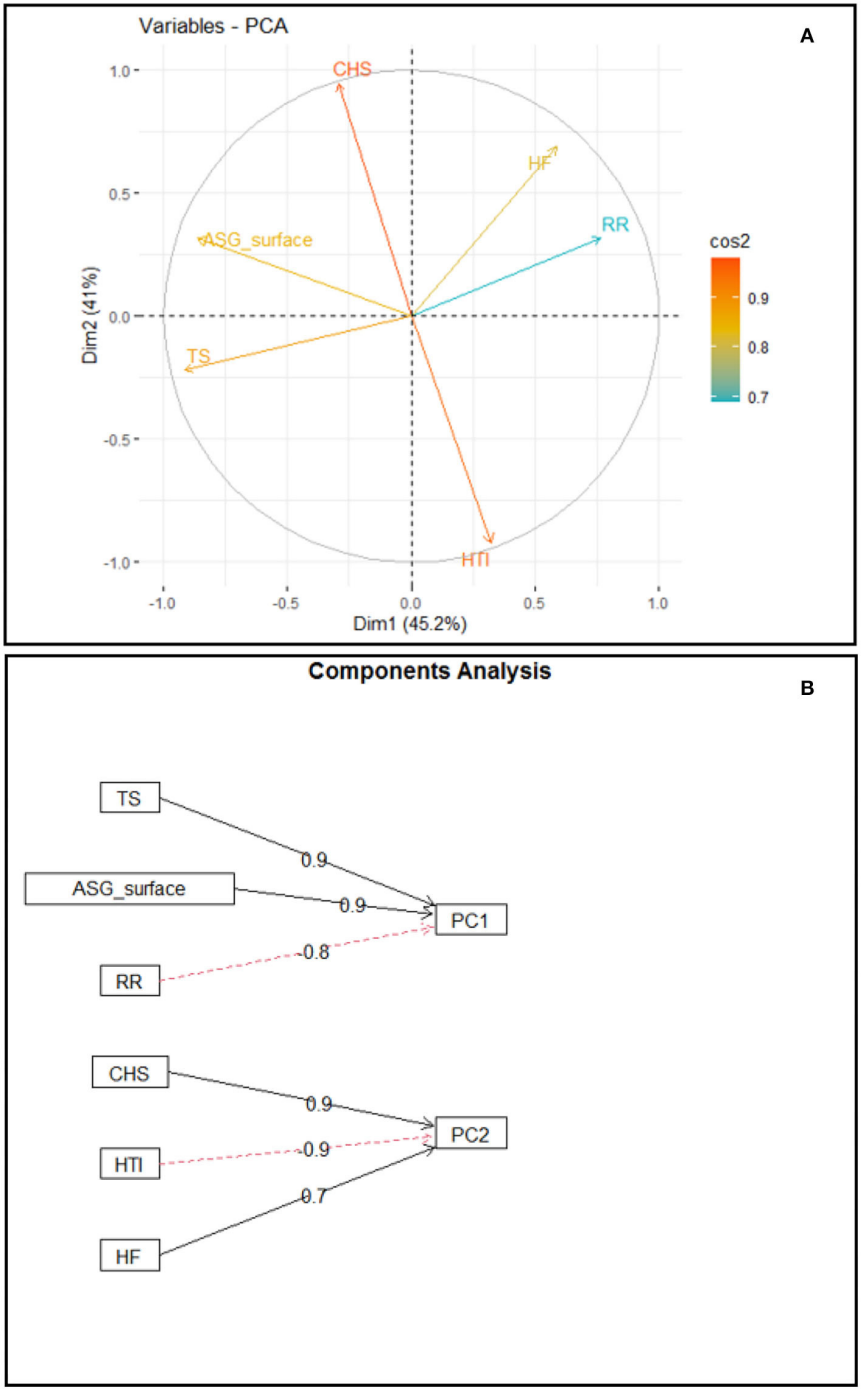
Biplot of PC1 × PC2, indicating the contribution of the variables in the dimensions **(A)**; Weight of the contribution of variables TS, ASG_surface, and RR to PC1 and weight of the contribution of variables CHS, HTI, and number of HF to PC2 **(B)**.

An analysis of the contribution of the variables in the dimensions and their respective weights shows that the variables that most contributed in dimension 1 were RR (0.766), TS (−0.914), and ASG_surface (−0.865), while the variables that most contributed to dimension 2 were HTI (−0.920), CHS (0.944), and HF (0.686) (Figure 4).

**Figure 5:**
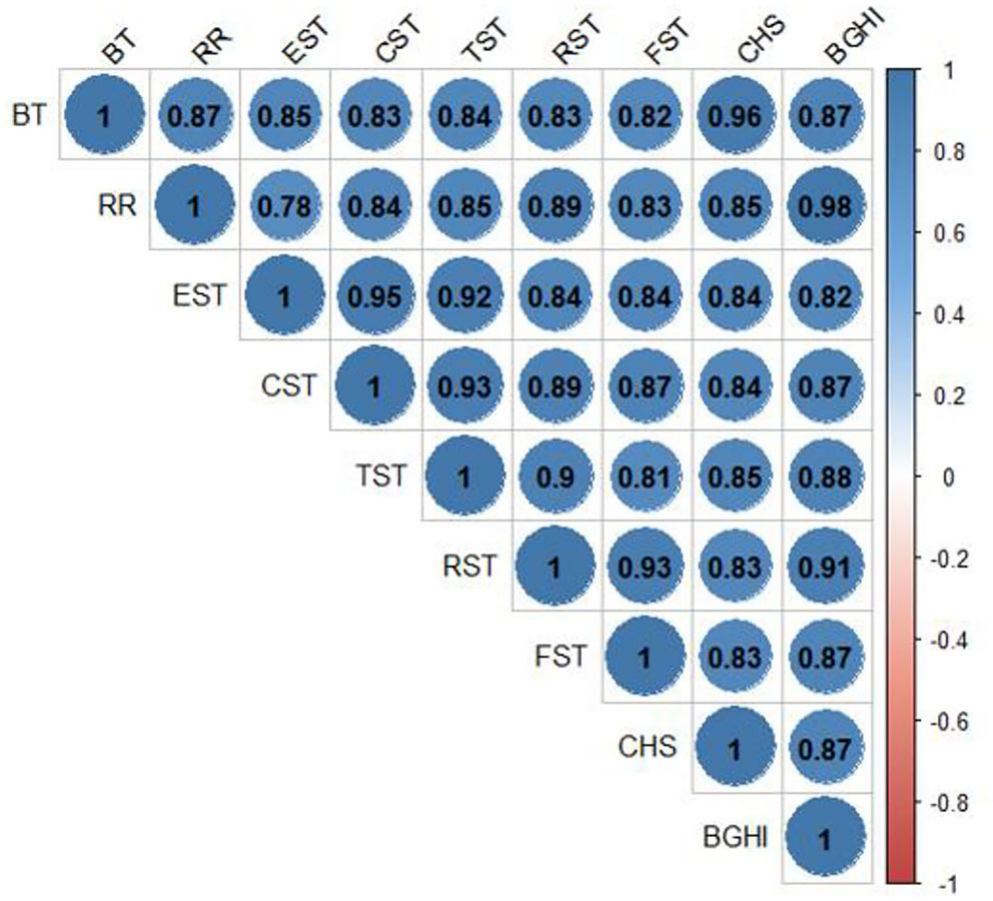
Linear correlations between the BGHI and internal BT, RR, EST, CST, TST, RST, FST, and CHS during the HTT performed in adult female buffaloes reared in humid tropical climate.

The correlations among internal body temperature, respiratory rate, body STs, stored heat, and the BGHI are presented in Figure 5. Analysis of the coefficients of Pearson's correlations showed significant positive correlations (*p* < 0.0001) for all pairs of these variables.

**Figure 6:**
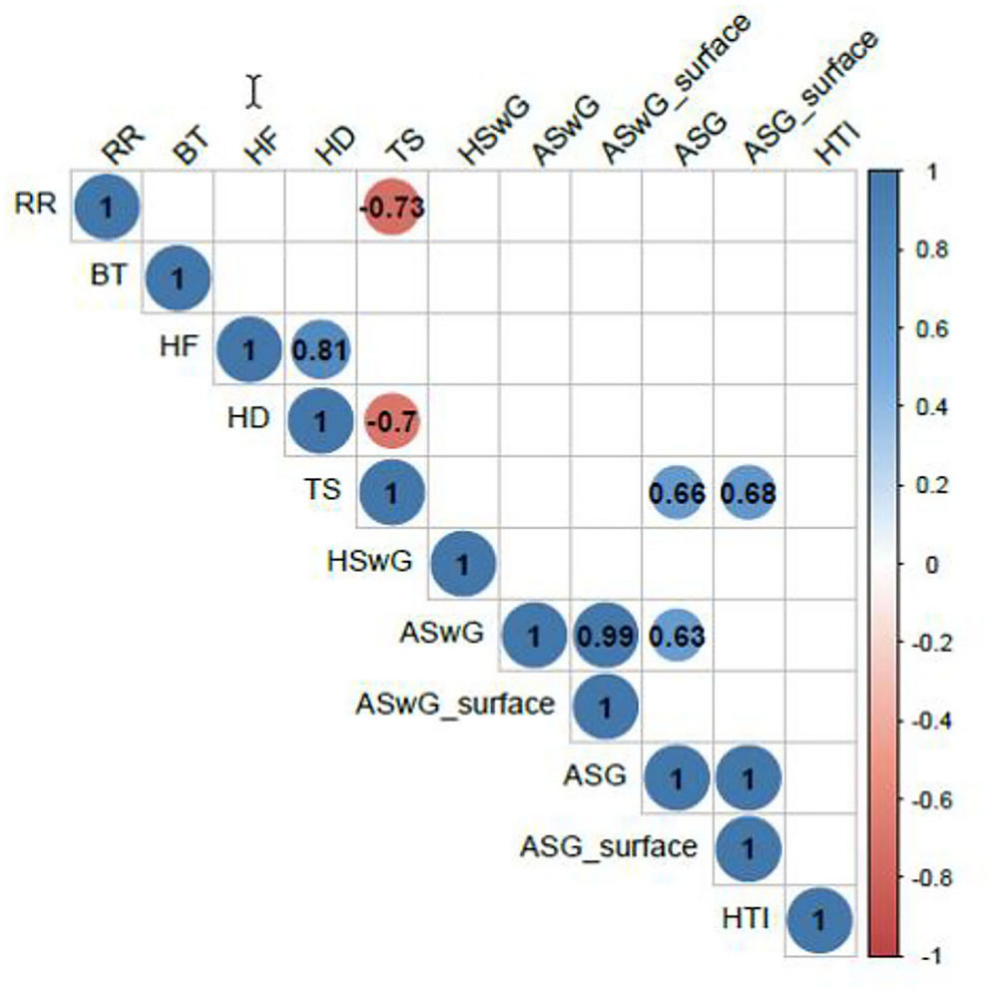
Linear correlations among the HTI, internal BT, and RR and the histomorphometric variables number of HF, hair density (HD), TS, HSwG, ASwG, ASwG_surface, ASG, and ASG_surface of adult female buffaloes reared in humid tropical climate.

The correlations among histomorphometric variables, the heat tolerance index, internal body temperature, and RR are presented in Figure 6. An analysis of the values of the linear coefficients showed a significant negative correlation (*p* = 0.0157) between RR and skin thickness and no significant correlation with internal body temperature.

## Discussion

The water buffaloes exhibited very high heat tolerance and used thermal polypnea as the main heat dissipation pathway. Their mean skin thickness observed was 6.03 ± 1.16 mm and, for the first time, the morphometry of sweat glands and sebaceous glands of buffaloes was performed, with a histological approach that allowed indicating its secreting activity. The PCs analysis enabled identifying morphological and functional associations relevant for thermoregulation of buffaloes, such as the interrelation of TS with the proportion of ASG and with respiratory rate. The study also showed that the buffaloes exhibited a high positive correlation between eyeball temperature and internal body temperature, a characteristic that may be explored in the future for the development of non-invasive diagnosis resources and remote monitoring of animal health and well-being.

The data in this study were collected during November and December, during the less rainy season of the Amazon biome, which goes from July to December (46). Although there was no significant difference in the dry-bulb temperature and relative air humidity values between the experimental periods, it was seen that the animals were in an environmental condition susceptible to heat stress since the mean air temperature values were above 32°C in all periods over the HTT.

The animals were exposed to environmental temperatures above the thermal comfort zone, characterized as an air temperature range in which the animal may exhibit its minimum metabolic rate and where homeothermy regulation is performed *via* exchange of sensible heat with the environment (61). According to Goswami and Narain (62), the thermoneutral zone for the water buffaloes ranges from 15.5°C to 21.2°C, while Shafie (63) places such zone between 13.0°C and 24.0°C. These authors suggest that the critical high temperature for buffaloes is above 29°C and 23.6°C, respectively, indicating that the animals in this study had to activate their heat dissipation pathways to maintain homeostasis as the lowest air temperature to which they were exposed was 32.14°C in the beginning of the HTT (Period 0).

Despite the high air temperatures during the tolerance test, relative air humidity remained with minimum values of 48% in Period 1 and maximum values of 56% in Period 0, staying within the relative humidity range considered optimal for most domestic species, which ranges from 40 to 70% (64). That can be explained by the time of day recommended for the application of the heat thermotolerance test, which coincided with moments of lower relative humidity in the circadian cycles occurring in the humid tropical climate subtype (46).

In this study, the classification of the BGHI indicates a danger situation (54), even when the animals were kept in the shade, with BGHI above 81. When the animals were exposed to sunlight, to identify their heat acquisition under a condition of direct solar radiation, the BGHI indicated an emergency situation (54), with maximum value of 101, since during that period the maximum black globe temperature value recorded was 52.1°C. The environmental thermal challenge situation for the animals is therefore evident, compelling them to activate their thermolysis pathways, an effect demonstrated by the alterations in physiological and biophysical variables monitored.

The relative air humidity recorded during the HTT favored heat exchange through respiratory evaporation, demonstrated by the pronounced increase in RR when the animals were under direct solar radiation, reaching maximum values of 116 mov·min^−1^. The increase in RR is an essential thermoregulatory response to maintain homeothermy and is used by buffaloes as a proactive thermolytic response to prevent hyperthermia in heat challenge situations (65).

The increase or decrease in RR depends on the intensity and duration of the heat stress to which the animals are submitted (66). This study showed the importance of heat loss through the respiratory pathway for buffaloes to maintain homeothermy. The pronounced reduction in RR occurred as soon as the animals returned to the shade after the heat challenge period (81–22 mov·min^−1^) confirms the rapid tolerance recovery of buffaloes after a heat stress situation, which reflected in a reduction by 0.58°C in internal body temperature. Several studies have described this rapid recovery of buffaloes in comparison with cattle (14, 67, 68).

When the animals were kept in a thermal comfort environmental condition provided by shade, their RR decreased and returned to values considered normal, with no significant difference among Periods 0, 2, and 3. According to Shafie (63), the RR of buffaloes ranged from 18 to 30 mov–min^−1^ for animals in thermal comfort. Thus, the importance of shade to maintain or favor homeothermy in buffaloes is evident. Indeed, recent reports indicate that the natural shade provided by silvopastoral systems contributes to the buffalo's well-being, to the expression of their normal behavior, and to increased performance (39). In tropical regions, providing shade becomes a requirement as it helps decrease excess direct irradiation received by the epidermis of buffaloes through direct solar radiation, particularly during the hottest hours of the day (61). Other studies carried out in the Amazon region have also indicated beneficial effects of shade on the physiological responses of buffaloes (69–75). In addition to seeking shade to rest and facilitate heat dissipation, buffaloes have the habit of wallowing (76). With this behavior, the animals lay on mud to favor body cooling *via* conduction, combined with the effects of convection caused by the wind and evaporation of the water in the mud (19). Since in this study, the animals were kept standing during the thermotolerance test to avoid interferences in the determination of HS, the effect of wallowing on body thermoregulation was not assessed.

This study recorded temperatures of eyeball surface, cheek, and forelimb, due to the greater microvascular blood flow of those anatomical regions (77), in addition to the trunk and rump, as those regions are more susceptible to oscillations in environmental conditions (78), particularly of solar radiation incidence. An assessment of the body STs when the animals were exposed to direct solar radiation showed a more significant increase in rump temperature (34.18–40.66°C) followed by increases in forelimb, trunk, cheek, and eyeball temperatures in relation to the baseline period. It is known that the epidermis temperature of the animal is influenced by the thermal condition of the environment, changes in blood flow, fur characteristics, and intensity of the skin latent thermolysis pathway (79). The increases in rump and trunk temperatures are directly related to environmental conditions in Period 1, when the daily irradiation level is historically maximum in the region (46), associated with morphofunctional characteristics of the epidermis of buffaloes, which favor heat acquisition. The epidermis of buffaloes has a high melanin concentration, which provides them heightened protection against ultraviolet radiation, however, with high absorbance (11). Moreover, the number of HF in the body surface makes them more susceptible to visible and infrared radiations, which are absorbed at higher intensity due to the color of the epidermis (10).

The increase in forelimb and cheek temperatures likely occurred due to the peripheral vasodilation in those regions. When an animal is exposed to heat stress, the initial thermoregulatory response that is set off is peripheral vasodilation (61, 79). In addition, considering the body area/mass ratio, greater blood flow is observed in the skin of the limbs and head than in the other regions of the body, which can be considered a thermoregulatory strategy for exchange of sensitive heat through radiation, conduction, and convection in those regions (77). It was found that only those two anatomic regions did not exhibit significant differences for STs recorded in Periods 0 and 3, which confirms vasodilation enables increased loss of sensitive heat, resulting in a reduction in forelimb and cheek temperatures when the animals were in environmental thermal comfort condition.

The orbital region has sympathetic fibers of the face nerve that innerve the capillary vessels of the facial and infraorbital artery, which respond to stressful stimuli (19). It was found that the lower alteration in STs between Periods 0 and 1 occurred in the eyeball, with an increase by only 3.82 °C. Such lower alteration may be related to the orbital anatomy, since, according to Casas–Alvarado et al. (80), adrenergic sympathetic fibers are sensitive to the neurosecretion of epinephrine and norepinephrine, which promotes vasoconstriction of capillaries, thus reducing the heat exchange rate and serving as a local thermoregulating mechanism.

An analysis of linear correlations showed that, among the body STs assessed, eyeball temperature exhibited the highest correlation with internal BT (*r* = 0.84523, *p* < 0.0001). The orbital region more closely corresponds to the internal BT since it suffers less alteration relative to the effects of environment temperature (81). Such results corroborate those found by Brcko et al. (50) and Barros et al. (43), who assessed the domestic buffaloes reared in the Amazon region. Bertoni et al. (82) also concluded that temperatures of the orbital region, of the nasal septum, and of the vulva have proved efficient in evaluating the thermal comfort of female buffaloes.

Pearson correlation between the variables studied and RR showed a greater positive correlation with the STs of the rump (*r* = 0.88598, *p* < 0.0001) and trunk (*r* = 0.84612, *p* < 0.0001) as those anatomic regions are more prone to temperature alterations when the animal is exposed to direct solar radiation (82). The negative correlation between RR and skin thickness (*r* = −0.73371, *p* = 0.0157) makes the animal spend less energy to dissipate heat since its RR is not as intense. Such characteristic observed is favorable to the animal and its productivity since altering the RR and depth results in a deviation of energy to respiration, which could be used in metabolic processes (79) such as growth, maintenance of gestation, or milk production.

The greater thermal gradient between the composite ST and air temperature measured in a dry-bulb thermometer was recorded in Period 1, with ΔT = 7.29°C. That greater gradient facilitates the dissipation of metabolic heat accumulated through the sensitive pathway and, due to the relative air humidity recorded, it also favored heat losses through the latent pathway, demonstrated by the alteration in respiratory rate. When the animals returned to a thermal comfort environment (Periods 2 and 3), a gradual reduction was observed in the calculated values of HS. Shade enabled a reduction by 0.0427 W·m^−1^ h^−1^ in stored heat, which reflected in a difference of 0.31°C in internal BT when compared with the internal temperatures measured between Periods 0 and 3. According to Pereira et al. (65), when in a thermal comfort condition, buffaloes exhibit a notable loss of body heat, provided by the reduction in stored heat.

Given the results of the tolerance index, the animals were classified as individuals of very high heat tolerance, with HTI values above 9.51 (45). Such thermotolerance capacity is directly related to the rapid physiological recovery of the animals after the heat stress situation due to the activation of their latent and sensitive thermolysis pathways. That result corroborates the findings by Pantoja et al. (75), who evaluated buffaloes reared in the Amazon region, in a humid tropical climate, indicating high heat tolerance in individuals from herds of that region.

A search of the scientific literature on the tegumental structures of buffaloes shows a lack of information and, at times, some controversies. Therefore, this study brings a valuable contribution regarding the skin histomorphometry of domestic buffaloes. The most striking characteristic of buffalo skin is the thickness of the epidermis, since the stratum corneum is more eminent and can be twice as thick than in cattle (14). The results from this study indicate that the mean skin thickness was 6.03 mm, corroborating the values reported by Ermetin (83), who determined a variation in skin thickness of adult domestic buffaloes of 6.0–7.6 mm. The findings also agree with the results found by Taneja and Bhatnagar (84), who indicate skin thickness of female Murrah buffaloes between 6.0 and 6.4 mm.

In addition, several recesses characterized by the extension of countless papillae in the dermis are found in the interface between the epidermis and dermis (14). According to Hafez and Anwar (16), those papillae have an important thermoregulatory role as the epidermis, when expanding into the dermis, changes the distribution of blood vessels. Thus, the arteries branch out more frequently, originating countless arterioles and capillaries, which favor heat dissipation *via* vasodilation. That effect can be observed in this study through the increase in ST of the animals when exposed to direct solar radiation, which was made evident by the results obtained with the use of infrared thermography.

Domestic buffaloes are animals that stand out for their low hair density (18). The number of hairs per unit of area decreases with age, making the adult animals nearly glabrous (85). This study found mean density of 2.00 HF per mm^2^, matching the results by Debbarma et al. (20) and Raheem, Elias and Ahmed (18), who indicate hair density in buffaloes of 1.91 and 2.78 HF per mm^2^, respectively. However, the number of HF in the literature varies, with values ranging from 91.84 cm^−2^ for Murrah animals (86), 135–145 cm^−2^ for Mediterranean buffaloes (17), and 394 cm^−2^ for Egyptian buffaloes (15). In fact, the lower hair density of buffaloes may have a double effect. It may facilitate heat dissipation by convection and evaporation, but it also reduces thermal insulation as the skin is more exposed to direct solar radiation (61).

In this study, the mean epithelium height of the sweat gland was 16.20 μm, with variation between 12.00 and 19.00 μm. The mean areas of the sweat and sebaceous glands were 17,283.92 μm^2^ and 11,821.95 μm^2^, respectively. The percentage of active sebaceous gland tissue was 1.08 ± 0.39%. In turn, the percentage of active sweat gland tissue in the sample analyzed was 1.57 ± 0.38%. That variable represents the ratio between the sum of the areas of the lumen of sweat glands identified in an image and the total area of tissue evaluated in the histological cut. Since the lumen of a sweat gland depends on it being filled by the sweat produced (79), the higher the percentage, the greater gland activity will be, representing the functionality of the sweat gland. Thus, the greater the gland activity at a given moment, the higher the possibility of the sweat produced being secreted, favoring the reduction of the skin ST. To the best of our understanding, such information is a first since no reference values were found in the literature to compare those variables. The recent studies have assessed the type and diameter of sweat (20) and sebaceous (21) glands in different regions of the body of buffaloes, in addition to their distribution (86); however, with no mention of the percentage of gland area, which is an indicative of activity of the respective glands.

Given the determination of the number of PCs, it was found that the first two PCs generated based on this analysis have eigenvalues >1 (λ*i*> 1) (87). The eigenvalues observed, of 2.709 for Dimension 1 and 2.458 for Dimension 2, accounted for 86.2% of the total variance in the dataset. Thus, the first two PCs effectively summarize the total sampling variance and may be used to study the set of those data. The weights of the variables that most contribute to Dimension 1 and the relationship between the variables and their placements along PC1, which models 45.2% of the variance of the data matrix, show that RR has an opposite sign to skin thickness and to the activity of sebaceous gland tissue. Such relationship is in accordance with the thermolytic response of buffaloes since skin thickness may condition the amount of energy that will be transferred to the core region of the body of the animal. The thicker the skin, the greater the protection against body overheating *via* reduction of thermal conductivity (88).

Moreover, the association of greater epidermis thickness with larger percentage of active sebaceous gland tissue favors the reduction in radiant thermal load absorbed by the skin. That occurs due to the greater reflectance of solar radiation in consequence of lipid secretion of the sebaceous glands, which spreads over the epidermis (10, 89), thus leading to a lower need of latent thermolysis and activation of thermal polypnea. Therefore, it is supposed that the first PC1 models the behavior of the preference of thermolytic pathway in buffaloes.

An analysis of the relationship between variables and their placements along PC2, which models 41.0% of the variance in the data matrix, showed that the HTI has an opposite sign to stored heat and number of HF. The relationship of those variables in the PC corresponds to the thermolytic responses of buffaloes since the heat stored along the day results in an increase in internal body temperature, leading to a lower heat tolerance index. The opposite relationship between the HTI and the number of HF demonstrates the preference of buffaloes in using latent thermolysis through the respiratory pathway in contrast with the cutaneous one. According to Marai and Haeeb (10), buffaloes have lower efficiency in losing heat through cutaneous pathways and the respiratory pathway is very relevant in dissipating endogenous heat to maintain homeothermy. A shortcoming of this study is that the local microclimate conditions, particularly the high relative air humidity, did not favor the direct quantification of sweat production by the standard technique, which uses paper disks impregnated with cobalt chloride (90). Therefore, it would be interesting to replicate the experimental protocol under climate conditions other than humid tropical climate. Furthermore, the thermoregulatory associations measured in this study were limited to young females and could be assessed in animals in other age groups and physiological conditions, such as calves and lactating or pregnant females.

## Conclusions

The quantification of the dynamic responses to the HTT and of the morphofunctional characteristics of buffalo tegument, such as the activities of sweat and sebaceous glands, hair density, and skin thickness, are the resources that allow measuring the thermotolerance capacity of buffaloes in a humid tropical environment. Buffalo skin thickness influences the conformation of dermal papillae and the distribution of peripheral blood vessels, which are identifiable *via* infrared thermography and whose participation in thermal exchanges can be successfully monitored in regions such as eyeball, cheek, trunk, rump, and forelimb. In humid tropical climate conditions, water buffaloes are able to quickly reduce their internal BT when protected from direct solar radiation, with the activation of thermal polypnea. In addition, eyeball ST can be used as a non-invasive indicator of thermal comfort of buffaloes as it exhibits a high correlation with internal body temperature.

### Nomenclature: Resource Identification Initiative

FLIR: FLIR tools, RRID:SCR_016330; HOBO: HOBOware Pro, RRID:SCR_021915; Leica Microsystems: Leica Microsystems, RRID:SCR_008960; Software Image J: ImageJ, RRID:SCR_003070; SAS: Statistical Analysis System, RRID:SCR_008567; R Studio Team: R Project for Statistical Computing, RRID:SCR_001905.

## Data Availability Statement

The raw data supporting the conclusions of this article will be made available by the authors, without undue reservation.

## Ethics Statement

The study was carried out in accordance with the Brazilian Guideline for Care and Use of Animals in Teaching or Scientific Research Activities. The procedures were approved by the Animal Bioethics Commission of Embrapa Eastern Amazon (Protocol 00715) and the results were reported according to the ARRIVE Guidelines: Animal Research Reporting in vivo Experiments.

## Author Contributions

RV, JL, MJ, and AG: conceptualization. RV, JL, MJ, AB, and AG: methodology. RV, AB, and AG: data curation. MP, MJ, JL, RV, and CO: investigation. AB and AG: formal analysis. RV and AG: writing the original draft. JL and AG: funding acquisition. MJ, JL, and AG: reviewing, and editing. AG: project administration, supervision. All authors have read and agreed to the published version of the manuscript.

## Funding

This study was funded in part by the Federal University of Pará, Coordenação de Aperfeiçoamento de Pessoal de Nível Superior (CAPES) Brasil (Finance Code 001), and Brazilian Agricultural Research Corporation–Embrapa (Precision Agriculture Network, Grant# 11.14.09.001.03.03). In addition, this study received financial support for the publication fee from the Pró-Reitoria de Pesquisa e Pós-Graduação (PROPESP/UFPA); ARG is the fellow of CNPq—National Council for Scientific and Technological Development.

## Conflict of Interest

MJ and AG were employed by Brazilian Agricultural Research Corporation. The remaining authors declare that the research was conducted in the absence of any commercial or financial relationships that could be construed as a potential conflict of interest.

## Publisher's Note

All claims expressed in this article are solely those of the authors and do not necessarily represent those of their affiliated organizations, or those of the publisher, the editors and the reviewers. Any product that may be evaluated in this article, or claim that may be made by its manufacturer, is not guaranteed or endorsed by the publisher.
